# Identification of Psychological Treatment Dropout Predictors Using Machine Learning Models on Italian Patients Living with Overweight and Obesity Ineligible for Bariatric Surgery

**DOI:** 10.3390/nu16162605

**Published:** 2024-08-08

**Authors:** Serena Marchitelli, Cristina Mazza, Eleonora Ricci, Valentina Faia, Silvia Biondi, Marco Colasanti, Alessandra Cardinale, Paolo Roma, Renata Tambelli

**Affiliations:** 1 UOC of Endocrinology, Metabolic Diseases, Andrology—CASCO (Center of High Specialization for the Treatment of Obesity), Policlinico Umberto I, Sapienza University of Rome, 00161 Rome, Italy; serenachiara.marchitelli@gmail.com; 2 Department of Dynamic and Clinical Psychology, & Health Studies, Sapienza University of Rome, Via degli Apuli 1, 00185 Rome, Italy; renata.tambelli@uniroma1.it; 3 Department of Neuroscience, Imaging and Clinical Sciences, University “G.d’Annunzio”, 66100 Chieti-Pescara, Italy; eleonora.ricci@unich.it; 4 The Free Spirit Collective Polyclinic, Dubai 252330, United Arab Emirates; valentina.faia@gmail.com; 5 Department of Human Neuroscience, Sapienza University of Rome, 00185 Rome, Italy; silvia.biondi@uniroma1.it (S.B.); paolo.roma@uniroma1.it (P.R.); 6 Department of Psychological, Health and Territorial Sciences, University “G.d’Annunzio”, 66100 Chieti-Pescara, Italy; marco.colasanti@unich.it; 7 National Institute of Nuclear Physics (INFN), 00186 Rome, Italy; alessandra.cardinale@uniroma1.it

**Keywords:** dropout, assessment, obesity, overweight, weight-loss surgery

## Abstract

According to the main international guidelines, patients with obesity and psychiatric/psychological disorders who cannot be addressed to surgery are recommended to follow a nutritional approach and a psychological treatment. A total of 94 patients (T0) completed a battery of self-report measures: Symptom Checklist-90—Revised (SCL-90-R), Barratt Impulsiveness Scale-11 (BIS-11), Binge-Eating Scale (BES), Obesity-Related Well-Being Questionnaire-97 (ORWELL-97), and Minnesota Multiphasic Personality Inventory-2 (MMPI-2). Then, twelve sessions of a brief psychodynamic psychotherapy were delivered, which was followed by the participants completing the follow-up evaluation (T1). Two groups of patients were identified: Group 1 (*n* = 65), who fully completed the assessment in both T0 and T1; and Group 2-dropout (*n* = 29), who fulfilled the assessment only at T0 and not at T1. Machine learning models were implemented to investigate which variables were most associated with treatment failure. The classification tree model identified patients who were dropping out of treatment with an accuracy of about 80% by considering two variables: the MMPI-2 Correction (K) scale and the SCL-90-R Phobic Anxiety (PHOB) scale. Given the limited number of studies on this topic, the present results highlight the importance of considering the patient’s level of adaptation and the social context in which they are integrated in treatment planning. Cautionary notes, implications, and future directions are discussed.

## 1. Introduction

Obesity and overweight, as forms of malnutrition, have reached epidemic levels, with over 1.9 billion adults with overweight in 2016, including more than 650 million people with obesity, and leading to over 4 million deaths in 2017. These conditions are linked to around 5 million deaths annually due to comorbidities like cardiovascular disease, diabetes, cancer, neurological disorders, respiratory diseases, and digestive disorders [1]. In 2016, the WHO reported that 58.5% of Italians aged 20 and over were people with overweight, with 19.9% with obesity. Recent data from the Italian National Statistics Institute show that 34.2% of Italians aged 18 and over are living with overweight, and 12% with obesity [2]. Obesity is a serious public health crisis impacting well-being and quality of life, often leading to social stigma. Its causes are multifaceted, involving genetic, metabolic, psychological, cultural, and social factors such as gender, education, socio-economic status, occupation, and lifestyle [3]. By achieving long-term weight loss and a resolution of associated comorbidities, bariatric surgery has proven to be an effective treatment for obesity, thereby increasing life expectancy [4,5,6]. As stated in the National Institute of Health (NIH) Consensus Conference Statement [7], candidates for bariatric surgery must be evaluated by a multidisciplinary team including medical, surgical, nutritional, and psychiatric experts. In 2006, a joint committee of the International Federation for the Surgery of Obesity and Metabolic Disorders (IFSO) and the European Association for the Study of Obesity (EASO) issued several recommendations for bariatric surgery procedures, covering indications, contraindications, preoperative evaluation, treatment, surgical techniques and follow-up, but also the assessment of possible psychopathologies, including alcoholism, drug addiction, psychotic disorders, major depression, and personality and eating disorders. Similarly, according to the Obesity Surgery Guidelines of the Italian Society of Bariatric and Metabolic Surgery (S.I.C.Ob) [8], the evaluation of a patient who intends to undergo bariatric surgery should not be limited to medical considerations alone, but should also include an analysis of the patient’s motivation, expectations, adherence to surgery, and the possible presence of mental disorders. In fact, in cases with established anamnestic data and/or clinical hypothesis, the S.I.C.Ob. guidelines recommend a psychiatric evaluation through clinical interviews and psychodiagnostic questionnaires to assess the existence of possible contraindications to the surgical treatment. If a contraindicative psychiatric diagnosis is formulated [8,9], patients might not be eligible for a bariatric pathway, and they are more likely to be addressed to a conservative approach to other types of endoscopic, nutritional, and anti-obesity drug/pharmacological strategies, but these alternatives cannot ignore a psychological support strategy [10] (Statement 19). Specifically, the S.I.C.Ob. Guidelines [8] identify absolute, major, and relative contraindications to bariatric surgery. Absolute contraindications for bariatric surgery include schizophrenia, non-compensating psychosis [11], and non-compensating bipolar disorder [12]. Bulimia nervosa (BN) is an absolute contraindication unless symptoms are fully remitted after psychotherapy. Major contraindications include substance abuse, alcohol dependence [13], suicide attempts, severe mental retardation, cognitive deficits, inadequate procedure understanding, and poor treatment adherence. Mood disorders and personality disorders are relative contraindications that can be reassessed after therapy. The binge-eating disorder (BED) [14] and night eating syndrome (NES) [15] require interdisciplinary assessment and psychotherapy. Anxiety and depression affect surgery outcomes but do not exclude it if managed with psychiatric support [16,17]. Therefore, it is important that patients adhere to psychological treatment, especially those diagnosed with anxiety, mood, and eating disorders, among others [18,19,20]. A large body of evidence, indeed, has demonstrated that individuals with obesity present higher rates of depression and anxiety, both at clinical and at subclinical levels, compared to normal-weight individuals [21,22].

Studies have identified predictors of dropout from obesity and weight-loss treatments [23], with rates of up to 80% [24]. Comparing results is difficult due to differences in study design, sample populations, clinical settings, treatment types, and definitions of dropout [25,26,27,28]. Predictors include demographics (e.g., BMI, employment, and smoking) [29], psychological health (e.g., depression, anxiety, and eating behaviors), personality factors (e.g., impulsiveness and social adjustment), and physical activity [23]. The aim of the present study was not to investigate the efficacy of psychotherapeutic treatment and/or the success of the alternative pathway in the case of ineligibility for bariatric surgery, but rather to investigate those characteristics and variables that may provide indications of patient attrition. Specifically, we hypothesized that demographic, anthropometric, and psychological characteristics of patients assessed as ineligible for bariatric surgery and with subclinical depressive–anxiety symptomatology might be predictors of dropout from alternative nutritional and psychotherapeutic treatment.

## 2. Materials and Methods

### 2.1. Participants

Participants were enrolled from patients accessing the C.A.S.C.O.—the High Specialization Center for the Care of Obesity at the University Hospital “Policlinico Umberto I”, Sapienza University of Rome, Italy. Patients with a body mass index (BMI) of ≥30 kg/m^2^ and/or metabolic disorders are eligible to undergo endocrinologic, cardiologic, internist, nutritional, and psychological screening at C.A.S.C.O. for in-day hospital treatments and to receive specific indications for treatment, which may consist of a nutritional diet, bariatric surgery (e.g., gastric bypass and sleeve gastrectomy), or endoluminal procedures. Body composition measurements included height (cm), weight (kg), and body mass index (BMI, kg/m^2^). Height was measured to the nearest millimeter using a mobile stadiometer (Seca 213, SECA Deutschland, Hamburg, Germany), with the participant’s head positioned in the horizontal Frankfort plane. Weight and other body composition variables were assessed using bioelectrical impedance analysis (BIA) with a Tanita BC-418 device (Tokyo, Japan), which is a non-invasive, cost-effective, and widely used method for evaluating body composition and clinical conditions. Data were collected between March 2021 and January 2023.

The inclusion criteria were as follows: age between 18 and 75 years; a body mass index (BMI) of ≥30 kg/m^2^; and ineligibility for bariatric surgery or endoluminal procedures due to reported psychiatric/psychological symptomatology during initial screening. The exclusion criteria were as follows: any reported major disease in the last 5 years; any reported or diagnosed actual inflammatory or autoimmune disease; corticosteroids for systemic use; any medication potentially affecting body weight or body composition; participation in a weight loss program in the last 3 months; renal failure; heart failure; history of viral or autoimmune liver disease or any other chronic liver disease; or excessive alcohol intake (≥140 g/week for men or 70 g/week for women).

Of the 182 patients identified, 111 agreed to the longitudinal research protocol, which consisted of a clinical and psychological assessment (T0), a brief psychotherapeutic treatment, and a follow-up evaluation (T1) (see Section 2.2). However, 17 participants did not complete the assessment at T0 and were excluded from the sample. Thus, the final sample included 94 participants, 71 females, and 23 males who were aged 18 to 73 years. Sixty-five of them completed the evaluation at both T0 and T1, whereas twenty-nine did not complete all twelve sessions of psychodynamic psychotherapy or did not return for follow-up evaluation (T1). The former constituted Group 1, the latter Group 2-dropout.

- Group 1 (*n* = 65) consisted of 47 females and 18 males (27.7%), with a mean age of 48.75 years. Regarding psychiatric and psychological disorders, 15 patients (23.1%) had a diagnosis of binge-eating disorder (BED), 1 patient (1.5%) had a diagnosis of Bulimia Nervosa Purging (BNP), 5 patients (7.7%) had a diagnosis of Eating Disorder Not Otherwise Specified (EDNOS), and 44 patients (67.7%) had minor emotional disorders (i.e., depression and anxiety at subclinical levels).
- Group 2-dropout (*n* = 29) consisted of 24 females and 5 males (17.2%), with an average age of 43.90 years. Regarding psychiatric and psychological disorders, 12 patients (41.4%) had a diagnosis of BED, 1 patient (3.4%) had a diagnosis of BED and NES (i.e., night eating syndrome), 2 patients (6.9%) had a diagnosis of EDNOS, and 14 patients (48.3%) had minor emotional disorders (i.e., depression and anxiety at subclinical levels). All patients were Caucasian. Descriptive statistics of the anthropometric variables of both groups are presented in Table 1.

The experimental procedure was approved by the local ethics committee (Board of the Department of Experimental Medicine, Faculty of Medicine and Dentistry, Sapienza University of Rome), and the guidelines detailed in the Declaration of Helsinki and the EEC Good Clinical Practice recommendations (Document 111/3976/88 of July 1990) were followed throughout this research.

### 2.2. Procedures

#### 2.2.1. Psychological Assessment (T0)

Regarding the psychological assessment (T0), patients were asked to complete the following self-report questionnaires: the Symptom Checklist-90-Revised (SCL-90-R), the Barratt Impulsiveness Scale-11 (BIS-11), the Binge-Eating Scale (BES), the Obesity-Related Well-Being Questionnaire (ORWELL-97), and the Minnesota Multiphasic Personality Inventory-2 (MMPI-2) (see Measures section). Table 2 shows the descriptive statistics of the psychological variables of Group 1 and Group 2-dropout.

#### 2.2.2. Psychotherapeutic Treatment

Subsequently, patients were sequentially assigned to one (out of six) volunteer-licensed psychotherapists trained in the psychotherapy protocol in a round-robin fashion. The first patient was assigned to Therapist 1, the second to Therapist 2, etc., until the sixth therapist was reached. This cycle was repeated until all 94 patients had been allocated. Based on this rotation, Therapists 1, 2, 3, and 4 received 16 patients each, while therapists 5 and 6 received 15 patients each. The psychotherapy protocol [30,31,32] included an initial assessment with 4 sessions of Brief Psychodynamic Investigation (BPI) aimed at making a diagnosis on personality functioning, and then short-term psychodynamic psychotherapy treatment of 8 sessions. In total, every participant had 12 sessions (4 BPI and 8 psychotherapy sessions). All sessions were individual, with each lasting 50 min.

The 4 sessions of BPI aimed to achieve the following: (a) formulate an initial psychodynamic hypothesis according to the patient’s core conflictual relationship theme and personality structure in order to define the patient’s problems and motivations for seeking therapy; (b) assess the patient’s capacity to invest in the therapeutic process and their motivation for seeking therapy by presenting the initial interpretation; (c) establish a therapeutic alliance; and (d) discuss the patient’s options and possible best interventions.

After these initial 4 sessions, the patients underwent an additional 8-session treatment. Specifically, short-term psychodynamic psychotherapy was provided on a psychoanalytic basis but with the new theoretical and practical contributions of modern approaches (primarily a vis-a-vis setting and a shorter duration). Specifically, the present protocol, based on Gilliéron’s [30,31,32] conceptualization, assesses the personality structure of the subject, assuming that the therapeutic relationship reflects the relational dynamics of daily life. Methodologically, much emphasis was placed on the first interview and on the relationship established between therapist and patient, focusing short-term intervention on the patient’s need for immediate relief and crisis resolution. In fact, this short-term psychodynamic psychotherapy aims to show the patient their symptoms, the context in which they occurred and their meaning, and the change they are going through. Each therapy was supervised monthly by a senior psychotherapist supervisor.

### 2.3. Measures

#### 2.3.1. Symptom Checklist-90-Revised (SCL-90-R)

The Symptom Checklist-90-Revised (SCL-90-R) [33] was administered to assess psychopathological symptoms that have occurred over the past week. The test consists of 90 items (e.g., “Nervousness or shakiness inside”) measured on a 5-point Likert scale, ranging from 1 (not at all) to 5 (very much). The scale includes 9 main psychological dimensions: somatization, obsessive–compulsive, interpersonal sensitivity, depression, anxiety, hostility, phobic anxiety, paranoid ideation, and psychoticism. The Italian validation was used in the present study [34].

#### 2.3.2. Barratt Impulsiveness Scale-11 (BIS-11)

The Barratt Impulsiveness Scale-11 (BIS-11) [35,36] was used to measure impulsiveness. The test consists of 30 items (e.g., “I plan tasks carefully”) rated on a 4-point Likert scale, ranging from 1 (rarely/never) to 4 (often/always). The scale allows for the detection of 3 components of impulsivity: attentional impulsivity, motor impulsivity, and non-planning impulsivity. The Italian version of the BIS-11 was used in the present study [37].

#### 2.3.3. Binge-Eating Scale (BES)

The Binge-Eating Scale (BES) [38] was administered to assess the presence of binge-eating episodes in a patient’s behavioral manifestation of eating and in the feelings and cognitions that characterize the episodes. The total score can range from 0 to 46; in fact, for each item (e.g., “I do not have any difficulty eating slowly in the proper manner”), there are 3 or 4 possible responses, each with a specific score. A total score below 17 indicates that the presence of binge-eating symptoms is unlikely; a total score between 17 and 27 identifies the possible presence of binge-eating symptoms; and a total score above 27 indicates the probable presence of binge-eating symptoms. In the present study, the Italian version of the BES was administered [39].

#### 2.3.4. Obesity-Related Well-Being Questionnaire (ORWELL-97)

To assess the quality of life (QoL) associated with obesity, the Italian version of the Obesity-Related Well-Being Questionnaire (ORWELL-97) [40,41] was adopted (e.g., “Does your weight hinder you in physical activity?”). The questionnaire has a 4-point Likert scale, ranging from 0 (not at all) to 3 (very much). A total scale score of 70 can be considered indicative of a significant influence of obesity on quality of life.

#### 2.3.5. Minnesota Multiphasic Personality Inventory-2 (MMPI-2)

The Minnesota Multiphasic Personality Inventory-2 (MMPI-2) [42] is a 51-scale self-report questionnaire that assesses personality and psychopathology. It consists of 567 items (e.g., “I certainly feel useless at times”) with dichotomous (true/false) response options. In the present study, the Italian version of the MMPI-2 [43,44] was administered, and the following scales were used for analysis: (a) the 3 main validity scales (F, L, and K), (b) the 10 standard clinical scales, and (c) the 15 content scales. The scores of the MMPI-2 scales were calculated according to the traditional method using the standard T scores (M = 50, SD = 10) [45]. The classification of MMPI-2 T scores is as follows: 55–60 = moderately high; 60–65 = high; and 65–70 = very high [42].

## 3. Statistical Analysis

Paired *t*-tests were conducted in Group 1, where the T-scores of each questionnaire at T0 and T1 were compared. The magnitude of the effect size was interpreted as follows: a d = 0.2 Cohen’s was considered indicative of a small effect, a d = 0.5 was a medium effect, and a d = 0.8 was a large effect [46]. The *p*-value was considered significant at the 0.05 level.

Chi-square tests were conducted to analyze any differences between Group 1 and Group 2-dropout in the variables related to sex, other psychiatric complications, the age of onset of overweight, and eating and nutritional disorders. To analyze any differences in the age, weight, height, and BMI, the scores obtained with the Mann–Whitney U test were instead compared. A Mann–Whitney’s U test was also performed to compare the T0-scale scores in the two groups.

These analyses were performed using SPSS v.28 software [47].

### Predictive Models

In this study, ML analyses were performed following a best practice workflow: (i) feature selection, (ii) model training and validation, and (iii) model testing in an out-of-group sample [48]. Since ML models are built to fit the data, it is important to test how the model fits the new/unseen data. For this reason, one part of the data (i.e., the training set) is generally used to train and validate the models, while another part (i.e., the testing set) is used to test the accuracy of the models on new examples. This procedure ensures generalization of the model and increases the replicability of the results [49,50,51]. For this purpose, participants were randomly divided into a training set, consisting of 70 participants (48 Group 1 participants and 22 Group 2-dropout participants), and a test set, consisting of 24 participants (17 Group 1 participants and 7 Group 2-dropout participants).

First, feature selection was performed with the goal of removing irrelevant features, which increase the generalization of the model by reducing the overfitting and noise in the data [52]. This procedure was performed using a feature selector based on Boruta’s algorithm [53], which selects the most statistically important features as they contribute the most to model performance. Boruta is an all-relevant feature selection wrapper algorithm that can work with any classification method that produces a variable importance measure (VIM). This method performs a top-down search for relevant features by comparing the importance of the original attributes with the randomly obtainable importance that is estimated using their permuted copies, progressively eliminating irrelevant features to stabilize the test. The predictors resulting from feature selection were provided as the input to the ML model used. The models were trained and validated through a 10-fold cross-validation procedure [54]. This consisted of repeatedly dividing the sample into training and validation sets. Thus, the sample of 70 participants (48 Group 1 participants and 22 Group 2-dropout participants) was randomly divided into 10 equal-sized subsamples or folds (i.e., 10 folds of 7 responses). Of the 10 subsamples, the data from only one subsample were kept as validation data to test the model, while the remaining 9 subsamples were used to generate training data. This process, using data from 70 participants, was repeated 10 times. In each iteration, one of the 10 subsets (or folds) was used as the validation dataset exactly once, while the remaining 9 subsets were used for training. The results of the 10 folds were then averaged to produce a single estimate of prediction accuracy [55]. Finally, to assess the accuracy of the validated models in classifying unseen participants as Group 1 and Group 2-dropout, they were tested on the out-of-sample test set (24 participants, of which 17 were from Group 1 and 7 from Group 2-dropout) [56]. We reported the predictive performance of the models based on the following metrics: accuracy, precision, recall, and F-measure (F1 score).

As mentioned above, we evaluated the accuracy of different ML classifiers to test whether the results were stable across classifiers and independent of specific model assumptions. Therefore, the chosen algorithms are representative of different classification strategies: logistic regression [57], random forest [58], and Naïve Bayes [59].

In addition, to facilitate the interpretability of the operations performed by the algorithms, we also implemented the classification tree algorithm as it allows us to interpret the operations performed and it highlights the logic of the classification [60]. Note that the algorithms were run using the default parameters set by R 4.2.1. Therefore, no parameter tuning was performed to increase the accuracy of the classification. These analyses were performed using R 4.1.2 software [61].

## 4. Results

### 4.1. Differences in Group 1 between T0 and T1

Between the first psychological assessment (T0) and the follow-up evaluation (T1), significant mean differences, with a medium effect’s magnitude, were found in the weight and BMI of Group 1 participants. In more detail, the mean weight (t(64) = 60.056, *p* < 0.001, d = 0.751) and BMI (t(64) = 5.777, *p* < 0.001, d = 0.717) were higher for T0 then T1.

Regarding the SCL-90-R scale, significant—albeit small—differences were found for the variables SOM (t(64) = 3.917, *p* < 0.001, d = 0.486); O-C (t(64) = 2.037, *p* = 0.046, d = 0.253); HOS (t(64) = 2.955, *p* = 0.004, d = 0.367); PHOB (t(64) = 2.536, *p* = 0.014, d = 0.315); PSY (t(64) = 2.304, *p* = 0.024, d = 0.286); and GSI (t(64) = 2.597, *p* = 0.012, d = 0.322), with the mean being higher at Time T0 than at Time T1.

Regarding the BIS-11 scale, only the BIS-11 M subscale had a significantly higher mean at Time T0 (t(64) = 2.687, *p* = 0.009), and the effect was small (d = 0.333).

**Figure 1:**
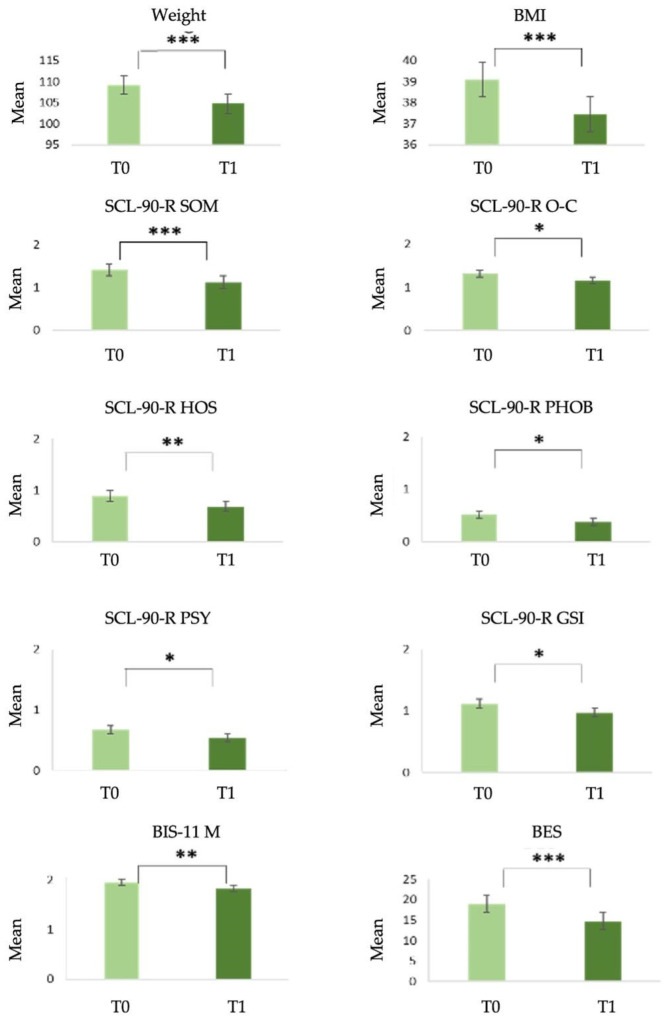
Statistically significant differences in Group 1 between T0 and T1. Note: * *p* < 0.05; ** *p* < 0.01; and *** *p* < 0.001. SCL-90-R, Symptom Checklist-90—Revised; BIS-11, Barratt Impulsiveness Scale-11; BES, Binge-Eating Scale; BMI, body mass index (kg/m^2^); and weight (kg).

Finally, the mean at T0 for the BES scale (t(64) = 4.054, *p* < 0.001) was also significantly higher, with a medium effect (d = 0.503). The results are shown in Table 3 and Figure 1.

### 4.2. Differences between Groups at T0

As shown in Table 4, there was no statistically significant association (*p* > 0.05) between the parameters and the groups, which were therefore homogeneous with respect to the considered characteristics.

Table 5 shows that there were no statistically significant differences (*p* > 0.05) between age, weight, height, BMI, and the groups, which were therefore homogeneous with respect to these characteristics.

In the SCL-90-R scale, it was observed that Group 2-dropout had statistically higher values than Group 1 for these variables: SOM (U = 681.500; *p* = 0.033); INT (U = 673.000; *p* = 0.027); DEP (U = 630.500; *p* = 0.011); PHOB (U = 620.000; *p* = 0.008); PSY (U = 681.000; *p* = 0.032); and GSI (U = 619.000; *p* = 0.008).

Regarding the BIS-11 scale, no statistically significant differences were found between the two groups.

BES scale values, on the other hand, were statistically higher in Group 2-dropout than in Group 1 (U = 696.000; *p* = 0.043), as were those on the ORWELL-97 scale (U = 555.000; *p* = 0.002).

Finally, in the MMPI-2 scales, statistically significant differences were found only for the variables 3-Hy (U = 641.000; *p* = 0.013) and DEP (U = 658.500; *p* = 0.020), and both the values were higher in Group 2-dropout than in Group 1.

### 4.3. Predictive Model

The 48 variables considered in the statistical analysis were included in the feature selection procedure. Among them, the following four were identified as the best set of predictors by the Boruta feature selector [53]: MMPI-2 K scale, ORWELL-97, SCL-90-R PHOB, and SCL-90-R PAR. The ML algorithms were trained, validated, and tested on these four variables according to the procedure described in Section Predictive Model. The results of the 10-fold validation procedure and the performance of the model [55] in the test set are shown in Table 6.

The classification accuracy remained stable among the different classifiers as it ranged from 71% to 83% in the test set. The best classifier was the classification tree, which achieved good accuracy in the training set (80%) and maintained a similar performance in the test set (79.17%).

To increase the transparency of the results, we report the rule used by the classification tree algorithm:

MMPI-2 K ≥ 51

| SCL-90-R PHOB < 0.515: Group 1

| SCL-90-R PHOB ≥ 0.515: Group 2-dropout

MMPI-2 K < 51: Group 1

It should be noted that the rule is very simple and allowed for the classification of responses with a 79% accuracy when we considered only two variables (i.e., the MMPI-2 K and SCL-90-R PHOB scales).

Finally, looking at the confounding matrix of the classification in the test set, for almost all algorithms, the specificity (i.e., the proportion of patients correctly predicted as Group 1 out of the total number of Group 1 patients observed) was higher than the sensitivity (i.e., the proportion of Group 2-dropout patients correctly predicted out of the total number of Group 2-dropout patients observed), except in Naïve Bayes. The following values were obtained—logistic regression: sensitivity = 0.57 and specificity = 0.94; random forest: sensitivity = 0.57 and specificity = 0.76; Naïve Bayes: sensitivity = 0.86 and specificity = 0.76; and classification tree: sensitivity = 0.71 and specificity = 0.82. Table 7 shows the test set classifications.

## 5. Discussion

According to the NIH and S.I.C.Ob. guidelines [7,8,9], patients with psychological and/or psychiatric disorders who are not candidates for bariatric surgery are recommended to follow an alternative approach consisting of nutritional and psychotherapeutic treatment, after which their eligibility for surgery is re-evaluated. In this longitudinal study, patients with obesity who were ineligible for bariatric surgery received brief psychodynamic psychotherapy along with nutritional treatment. To better understand which demographic, anthropometric, and psychological variables might be the best predictors of dropout from psychological treatment, artificial intelligence techniques were applied. The best classifier, trained using a 10-fold cross-validation procedure, was the classification tree model, which demonstrated good accuracy (around 80%). Specifically, the classification tree model enabled the classification of dropout and non-dropout responses by considering only the scores on the Correction (K) scale of the MMPI-2 and the Phobic Anxiety (PHOB) scale of the SCL-90-R. Interestingly, the machine learning algorithms did not identify psychological symptomatology as a predictive factor for dropout from psychotherapy. Instead, the results highlighted that the patient’s level of adjustment, their defensiveness (i.e., the MMPI-2 K scale), and the symptoms related to persistent and irrational fears associated with specific objects, situations, or activities that lead to avoidance behavior (SCL-90-R PHOB scale) were the best predictors in the present sample. Specifically, the Correction (K) subscale of the MMPI-2 assesses the individual’s tendency to deny a psychopathological condition and instead present a positive image of themselves. Indeed, a high score on this scale (T > 65) could indicate a defensive attitude on the part of the individual during the administration of the test. Furthermore, the K scale could be considered as a measure of the “denial” defense mechanism, which, like the other defense mechanisms, could be present both at a pathological level and as a result of an adaptive strategy [42,62]. This finding is in line with previous studies [63], which have found that the denial defense mechanism, together with projection, regression, and removal, is prevalent in patients with obesity. In detail, the denial defense mechanism is “an unconscious process that functions to resolve emotional conflict or reduce anxiety”, and it is one of the most primitive and immature defense mechanism in which “unpleasant thoughts, feelings, wishes, or events are ignored or excluded from conscious awareness” [64]. Moreover, patients included in the present study reported high scores on the Phobic Anxiety (PHOB) subscale of the SCL-90-R, which refers to “a persistent fear response to a specific person, place, object, or situation-that is recognized as irrational and out of proportion to the stimulus and leads to avoidance or escape behavior” [33], and it resulted in being a predictor of dropout from the present psychotherapeutic treatment. The result is consistent with the study by Jensen, Mortensen, and Lotz [65], which found that high scores on the Phobic Anxiety scale were significantly associated with dropout from psychodynamic psychotherapeutic treatment, and it consists of 39 sessions where the patients are diagnosed with severe anxiety. Furthermore, this aligns with previous studies showing that anxiety and depressive symptomatology, along with poor distress tolerance and eating disorder symptoms, may predict non-adherence to treatment prescriptions and, consequently, higher dropout rates from multidisciplinary therapies [66,67].

Given the limited number of studies on patients assessed as not eligible for bariatric surgery who undergo brief psychodynamic psychotherapy, at least two hypotheses could be formulated to explain the present results on dropout. Firstly, defensiveness, the presence of denial defense mechanisms, and the patients’ reported levels of adaptation to their social context could negatively affect the therapeutic alliance established during psychological treatment, resulting in higher dropout rates. Moreover, it could be assumed that experiencing an unsupportive and judgmental social context—due to the social stigma surrounding overweight and obesity conditions [68]—could lead individuals to avoid the social context itself. Consequently, such individuals might present themselves in an overly positive manner, denying aspects of maladaptation both personally and socially. They could also be patients who tend to avoid addressing specific issues directly or indirectly related to their relationship with food during psychotherapy sessions. Lastly, it should be noted that the sample consisted of patients who initially requested an assessment for bariatric surgery but were instead advised to follow a different weight loss intervention consisting of nutritional and psychotherapeutic treatment. Thus, the patients’ intrinsic motivation might not have been sufficient to fully adhere to the alternative treatment to bariatric surgery, leading to withdrawals from it.

Some limitations of the present study should be noted. First, the two groups differed in size, with only 29 patients in Group 2-dropout and 65 patients in Group 1, and the total sample size consisted of 94 patients. A further limitation concerns the brevity of the follow-up assessment, which was conducted after the conclusion of the twelve psychotherapy sessions. Therefore, the findings of this study should be generalized with caution. It must also be considered that, even though the results may provide initial evidence of the efficacy of the psychotherapeutic treatment on the psychological symptomatology reported by patients who completed the sessions, along with a reduction in weight and BMI, this was not the primary aim of this study. Consequently, no control group was enrolled, and no further analysis of treatment outcomes was conducted.

## 6. Conclusions

The results highlight the importance of considering the patient’s reported level of adjustment, the normalization of their condition, and avoidance of uncomfortable feelings, probably around food, and the extent to which the patient feels understood, non-judged, and supported by their social context. Therefore, future research should further explore the role of adjustment and social context in patients with obesity and incorporate these factors into treatment planning. Finally, future follow-ups are needed that consider the role of these variables in treatment withdrawal.

## Figures and Tables

**Table 1:** Descriptive statistics of the anthropometric variables at T0 of Group 1 and Group 2-dropout.

Variables		Group 1 (*n* = 65) M (SD)	Group 2-Dropout (*n* = 29) M (SD)	Min-Max
**Anthropometric Variables**	Weight (kg)	109.24 (27.01)	107.91 (15.37)	80–200
	Height (m)	1.67 (0.09)	1.66 (0.08)	1.49–1.92
	BMI (kg/m^2^)	39.11 (7.49)	39.32 (5.29)	27.40–60.38
	Age	48.75 (12.00)	43.90 (14.17)	18–75

Note: *n*, sample size; M, mean; SD, standard deviation; and BMI, body mass index.

**Table 2:** Descriptive statistics of the psychological variables at T0 of Group 1 and Group 2-dropout.

Variables		Group 1 (*n* = 65) M (SD)	Group 2-Dropout (*n* = 29) M (SD)	Min-Max
**SCL-90-R** **Psychopathological Symptoms**	Somatization (SOM)	1.41 (0.78)	1.80 (0.83)	0–3.25
	Obsessive–Compulsive (O-C)	1.32 (0.73)	1.58 (0.82)	0–3.10
	Interpersonal Sensitivity (I-S)	1.19 (0.80)	1.70 (1.04)	0–3.78
	Depression (DEP)	1.41 (0.82)	1.86 (0.78)	0–3.80
	Anxiety (ANX)	1.05 (0.63)	1.28 (0.75)	0–2.90
	Hostility (HOS)	0.89 (0.75)	1.16 (0.79)	0–3.17
	Phobic Anxiety (PHOB)	0.51 (0.58)	0.77 (0.50)	0–2.14
	Paranoid Ideation (PAR)	1.14 (0.75)	1.53 (0.96)	0–3.33
	Psychoticism (PSY)	0.67 (0.58)	1.01 (0.79)	0–3.60
	Global Severity Index (GSI)	1.12 (0.56)	1.49 (0.61)	0.17–2.97
**MMPI-2**	**MMPI-2 Validity scales**			
	Infrequency (F)	59.29 (10.96)	59.21 (10.02)	40–101
	Lie (L)	50.15 (7.88)	49.93 (9.86)	30–69
	Correction (K)	44.92 (8.27)	47.76 (10.60)	30–67
	**MMPI-2 Clinical scales**			
	Hypochondriasis (1-Hs)	64.37 (12.77)	65.38 (9.77)	36–91
	Depression (2-D)	62.88 (12.66)	67.17 (10.20)	40–90
	Hysteria (3-Hy)	59.02 (11.70)	65.00 (9.94)	34–85
	Psychopathic Deviate (4-Pd)	61.89 (13.26)	63.83 (7.72)	32–88
	Masculinity/Femininity (5-MF)	48.94 (11.52)	50.55 (12.05)	30–77
	Paranoia (6-Pa)	58.94 (11.57)	58.31 (9.74)	36–88
	Psychasthenia (7-Pt)	57.15 (11.76)	57.72 (9.44)	31–81
	Schizophrenia (8-Sc)	58.31 (10.64)	57.34 (8.39)	30–87
	Hypomania (9-Ma)	50.29 (10.02)	51.07 (10.60)	35–78
	Social Introversion (0-Si)	56.77 (10.27)	55.86 (12.09)	31–80
	**MMPI-2 Content scales**			
	Anxiety (ANX)	60.68 (11.98)	63.79 (10.23)	36–86
	Fears (FRS)	53.77 (11.53)	55.45 (10.05)	33–84
	Obsessiveness (OBS)	55.06 (11.23)	53.31 (9.95)	36–86
	Depression (DEP)	59.05 (11.30)	65.17 (11.15)	36–89
	Health Concerns (HEA)	63.77 (11.41)	64.07 (10.75)	47–103
	Bizarre Mentation (BIZ)	54.17 (9.03)	52.83 (9.93)	34–86
	Anger (ANG)	54.20 (12.40)	54.24 (13.49)	33–87
	Cynicism (CYN)	54.00 (10.48)	58.00 (13.72)	37–82
	Antisocial Practices (ASP)	49.89 (7.60)	50.52 (10.59)	33–80
	Type A (TPA)	52.42 (11.49)	49.07 (9.54)	32–84
	Low Self-Esteem (LSE)	58.80 (10.29)	57.93 (9.32)	40–88
	Social Discomfort (SOD)	55.14 (10.29)	53.97 (9.26)	37–83
	Family Problems (FAM)	58.25 (10.88)	56.79 (8.94)	42–87
	Work Interference (WRK)	58.08 (12.20)	57.31 (9.41)	39–84
	Negative Treatment Indicators (TRT)	57.74 (10.54)	59.17 (10.11)	35–82
**BIS–11** **Impulsivity**	Attentional Impulsivity (A)	1.90 (0.36)	1.98 (0.46)	1.12–3.00
	Non-Planning Impulsivity (NP)	2.43 (0.41)	2.50 (0.37)	1.09–3.27
	Motor Impulsivity (M)	1.96 (0.42)	2.09 (0.45)	1.54–3.27
	Impulsivity Total	2.10 (0.31)	2.20 (0.33)	1.43–2.90
**BES** **Binge-Eating Episodes**		19.00 (9.71)	23.66 (10.22)	1–42
**ORWELL-97** **Quality of Life**		69.27 (23.07)	85.78 (21.94)	26.0–120.5

Note: *n*, sample size; M, mean; SD, standard deviation; SCL-90-R, Symptom Checklist-90—Revised; MMPI-2, Minnesota Multiphasic Personality Inventory-2; BIS-11, Barratt Impulsiveness Scale-11; BES, Binge-Eating Scale; and ORWELL-97, Obesity-Related Well-Being Questionnaire.

**Table 3:** Paired *t*-test on weight, BMI, SCL-90-R, BIS-11, BES, and ORWELL-97 scales.

Variable		Mean	SD	Difference
**Weight** kg	T0 T1	109.24 104.73	27.00 26.43	**4.51**
**BMI** kg/m^2^	T0 T1	39.11 37.46	7.49 7.66	**1.65**
**SCL-90-R** Somatization (SOM)	T0 T1	1.41 1.13	0.78 0.76	**0.29**
Obsessive–Compulsive (O-C)	T0 T1	1.32 1.16	0.73 0.74	**0.15**
Interpersonal Sensitivity (I-S)	T0 T1	1.19 1.11	0.80 0.80	0.08
Depression (DEP)	T0 T1	1.41 1.37	0.82 0.86	0.03
Anxiety (ANX)	T0 T1	1.05 0.94	0.63 0.73	0.12
Hostility (HOS)	T0 T1	0.89 0.69	0.75 0.70	**0.21**
Phobic Anxiety (PHOB)	T0 T1	0.52 0.38	0.58 0.49	**0.13**
Paranoid Ideation (PAR)	T0 T1	1.14 1.09	0.75 0.75	0.05
Psychoticism (PSY)	T0 T1	0.67 0.54	0.58 0.50	**0.13**
Global Severity Index (GSI)	T0 T1	1.12 0.97	0.57 0.61	**0.15**
**BIS-11** Attentional Impulsivity (A)	T0 T1	1.90 1.84	0.36 0.45	0.06
Motor Impulsivity (M)	T0 T1	1.96 1.83	0.42 0.40	**0.13**
Non-Planning Impulsivity (NP)	T0 T1	2.44 2.45	0.41 0.47	−0.009
Impulsivity Total	T0 T1	2.10 2.07	0.31 0.32	0.03
**BES**	T0 T1	19.00 14.83	9.71 10.96	**4.17**
**ORWELL-97**	T0 T1	69.17 65.80	23.07 22.82	3.47

Note: Statistically significant effects (*p* < 0.05) are in bold. The final column reports the difference between the two means (T0-T1). M, mean; SD, standard deviation; BMI, body mass index; SCL-90-R, Symptom Checklist-90—Revised; BIS-11, Barratt Impulsiveness Scale-11; BES, Binge-Eating Scale; and ORWELL-97, Obesity-Related Well-Being Questionnaire.

**Table 4:** The chi-square results between the groups and gender, the other psychiatric complications, the age of onset of overweight, and eating and nutritional disorders.

Variable	Group 1 (*n* = 65)	Group 2-Dropout (*n* = 29)	*p*-Value
Gender			0.276
Male	18 (27.7%)	5 (17.2%)	
Female	47 (72.3%)	24 (82.8%)	
**Eating and nutritional disorder**			0.18
**BED**	**15 (23.1%)**	12 (41.4%)	
**BED and NES**	**0 (0%)**	1 (3.4%)	
**BNP**	**1 (1.5%)**	0 (0%)	
**EDNOS**	**5 (7.7%)**	2 (6.9%)	
**None**	**44 (67.7%)**	14 (48.3%)	
**Age of onset of overweight**			0.36
Under 6 years	20 (30.8%)	12 (41.4%)	
6–13 years	8 (12.3%)	7 (24.1%)	
14–19 years	12 (18.5%)	5 (17.2%)	
20–29 years	11 (16.9%)	1 (3.4%)	
30–39 years	5 (7.7%)	3 (10.3%)	
40–49 years	6 (9.2%)	1 (3.4%)	
50–59 years	2 (3.1%)	0 (0%)	
Over 60 years	1 (1.5%)	0 (0%)	
**Other psychiatric complications**			0.74
**Yes**	16 (24.6%)	6 (21.4%)	
**No**	49 (75.4%)	22 (78.6%)	

Note: *n*, sample size; BED, binge-eating disorder; NES, night eating syndrome; BNP, bulimia nervosa purging type; and EDNOS, Eating Disorder Not Otherwise Specified.

**Table 5:** Results of the Mann–Whitney U test on age, weight, height, BMI, SCL-90-R, BIS-11, BES, ORWELL-97, and MMPI-2 scales at T0.

Variable	Median Group 1 (*n* = 65)	Median Group 2-Dropout (*n =* 29)	Mann–Whitney U Test	*p*-Value
**Age**	50.00	46.00	1134.00	0.117
**Weight**	102.00	106.00	831.50	0.363
**Height**	1.65	1.65	961.00	0.879
**BMI**	36.65	36.98	855.00	0.474
**SCL-90-R**				
Somatization (SOM)	**1.42**	**1.92**	**681.50**	**0.033**
Obsessive–Compulsive (O-C)	1.30	1.50	762.50	0.140
Interpersonal Sensitivity (I-S)	**1.00**	**1.78**	**673.00**	**0.027**
Depression (DEP)	**1.38**	**1.92**	**630.50**	**0.011**
Anxiety (ANX)	1.00	1.20	799.00	0.239
Hostility (HOS)	0.67	1.00	745.50	0.106
Phobic Anxiety (PHOB)	**0.29**	**0.71**	**620.00**	**0.008**
Paranoid Ideation (PAR)	1.00	1.50	703.50	0.050
Psychoticism (PSY)	**0.60**	**1.00**	**681.00**	**0.032**
Global Severity Index (GSI)	**1.04**	**1.47**	**619.00**	**0.008**
**BIS**				
Attentional (A)	1.87	2.00	814.50	0.293
Motor (M)	1.90	2.09	801.00	0.246
Non-planning (NP)	2.45	2.54	890.50	0.670
Total	2.10	2.16	798.50	0.238
**BES**	**17.00**	**25.00**	**696.00**	**0.043**
**ORWELL-97**	**68.50**	**93.00**	**555.00**	**0.002**
MMPI-2 Validity Scales				
Infrequency (F)	59	59	915.00	0.822
Lie (L)	50	52	928.00	0.905
Correction (K)	46	51	784.50	0.195
**MMPI-2 Clinical Scales**				
Hypochondriasis (1-Hs)	62	65	865.00	0.525
Depression (2-D)	62	68	753.00	0.120
Hysteria (3-Hy)	**57**	**67**	**641.00**	**0.013**
Psychopathic Deviate (4-Pd)	63	63	873.50	0.572
Masculinity/Femininity (5-MF)	48	50	848.50	0.441
Paranoia (6-Pa)	59	58	987.50	0.712
Psychasthenia (7-Pt)	56	57	905.50	0.762
Schizophrenia (8-Sc)	55	57	983.50	0.737
Hypomania (9-Ma)	48	48	908.50	0.780
Social Introversion (0-Si)	59	57	1004.00	0.614
**MMPI-2 Content Scales**	59	64	778.00	0.177
Anxiety (ANX)				
Fears (FRS)	52	57	833.50	0.371
Obsessiveness (OBS)	51	51	984.50	0.730
Depression (DEP)	**56**	**67**	**658.50**	**0.020**
Health Concerns (HEA)	62	62	918.00	0.841
Bizarre Mentation (BIZ)	54	50	1018.00	0.535
Anger (ANG)	51	48	970.50	0.818
Cynicism (CYN)	51	60	825.50	0.338
Antisocial Practices (ASP)	49	48	979.00	0.765
Type A (TPA)	50	47	1109.50	0.171
Low Self-Esteem (LSE)	55	57	963.00	0.866
Social Discomfort (SOD)	53	52	1003.50	0.617
Family Problems (FAM)	57	53	1014.50	0.555
Work Interference (WRK)	54	59	905.00	0.759
Negative Treatment Indicators (TRT)	56	58	868.50	0.544

Note: The median, Mann–Whitney U test results, and *p*-value for all the anthropometric and psychological factors are reported. *n*, sample size; weight (kg); height (m); BMI, body mass index (kg/m2); SCL-90-R, Symptom Checklist-90—Revised; MMPI-2, Minnesota Multiphasic Personality Inventory-2; BIS-11, Barratt Impulsiveness Scale-11; BES, Binge-Eating Scale; and ORWELL-97, Obesity-Related Well-Being Questionnaire. Statistically significant *p*-values (*p* < 0.05) are in bold.

**Table 6:** Results of the different machine learning algorithms in the 10-fold cross-validation and in the test set.

Algorithm		Accuracy	Precision	Recall	F-Measure
**Logistic** **regression**	10-fold cross-validation	75.71%	0.958	0.754	0.844
	Test set	83.33%	0.800	0.571	0.666
**Random forest**	10-fold cross-validation	70%	0.813	0.765	0.788
	Test set	70.83%	0.500	0.571	0.533
**Naïve Bayes**	10-fold cross-validation	77.14%	0.958	0.767	0.852
	Test set	79.17%	0.600	0.857	0.706
**Classification tree**	10-fold cross-validation	80%	0.958	0.793	0.868
	Test set	79.17%	0.625	0.714	0.667

**Table 7:** Results of the confusion matrix in the test set.

		Reference	
	Predicted	Group 2-Dropout (*n* = 7)	Group 1 (*n* = 17)
Logistic regression	Group 2-dropout	4	1
	Group 1	3	16
Random forest	Group 2-dropout	4	4
	Group 1	3	13
Naïve Bayes	Group 2-dropout	6	4
	Group 1	1	13
Classification tree	Group 2-dropout	5	3
	Group 1	2	14

Note: *n*, sample size.

## Data Availability

The data presented in this study are available on request from the corresponding authors due to privacy.

## References

[1] World Health Organization (WHO) (2022). Who European Regional Obesity Report—2022. https://apps.who.int/iris/bitstream/handle/10665/353747/9789289057738-eng.pdf?utm_source=townandcountrytoday.com&utm_campaign=townandcountrytoday.com%3A%20outbound&utm_medium=referral.

[2] National Institute of Statistics (Istituto Nazionale di Statistica, ISTAT) Health Risk Factors: Smoking, Obesity, Alcohol, and Sedentary Lifestyle. https://www.istat.it/it/archivio/270163.

[3] Cockerham W.C. (2022). Theoretical Approaches to Research on the Social Determinants of Obesity. Am. J. Prev. Med..

[4] Guan W., Lin S., Fu Z., Yang N., Shen J., Liu R., Liang H. (2023). Five-Year Physical and Psychosocial Outcomes in Obese Adolescents with and without Metabolic Bariatric Surgery. J. Adolesc. Health.

[5] Arterburn D.E., Telem D.A., Kushner R.F., Courcoulas A.P. (2020). Benefits and Risks of Bariatric Surgery in Adults: A Review. JAMA.

[6] Fink J., Seifert G., Blüher M., Fichtner-Feigl S., Marjanovic G. (2022). Obesity Surgery: Weight Loss, Metabolic Changes, Oncological Effects, and Follow-Up. Dtsch. Arztebl. Int..

[7] National Institute of Health (NIH) (1991). NIH Conference: Gastrointestinal Surgery for Severe Obesity. Consensus Development Conference Panel. Ann. Intern. Med..

[8] Italian Society of Surgery of Obesity and Metabolic Diseases (Società Italiana di Chirurgia dell’Obesità e delle Malattie Metaboliche, S.I.C.Ob.) Obesity Surgery Guidelines (Linee Guida di Chirurgia dell’Obesità). https://www.sicob.org/00_materiali/linee_guida_2016.pdf.

[9] Italian Society of Surgery of Obesity and Metabolic Diseases (Società Italiana di Chirurgia dell’Obesità e delle Malattie Metaboliche, S.I.C.Ob.) The Surgical Treatment of Obesity and Associated Complications (La Terapia Chirurgica dell’Obesità e delle Complicanze Associate). https://www.sicob.org/00_materiali/Linee_Guida_SICOB_2023.pdf.

[10] Zappa M.A., Iossa A., Busetto L., Chiappetta S., Greco F., Lucchese M., Micanti F., Mingrone G., Navarra G., Raffaelli M. (2023). SICOB-endorsed National Delphi Consensus on Obesity Treatment Optimization: Focus on Diagnosis, Pre-operative Management, and Weight Regain/Insufficient Weight Loss Approach. Eat Weight Disord..

[11] Rius Acebes L., Sánchez-Pacheco-Tardon M., Orozco Beltrán D. (2024). ¿Cuándo derivar a endocrinología el paciente con obesidad? Indicaciones actuales de la cirugía bariátrica [When should a patient with obesity be referred to endocrinology? Current indications for bariatric surgery]. Aten. Primaria.

[12] McElroy S.L., Keck P.E. (2012). Obesity in Bipolar Disorder: An Overview. Curr. Psychiatry Rep..

[13] Saules K.K., Wiedemann A., Ivezaj V., Hopper J.A., Foster-Hartsfield J., Schwarz D. (2010). Bariatric Surgery History Among Substance Abuse Treatment Patients: Prevalence and Associated Features. Surg. Obes. Relat. Dis..

[14] Meany G., Conceição E., Mitchell J.E. (2014). Binge Eating, Binge Eating Disorder and Loss of Control Eating: Effects on Weight Outcomes after Bariatric Surgery. Eur. Eat. Disord. Rev..

[15] Colles S.L., Dixon J.B. (2006). Night Eating Syndrome: Impact on Bariatric Surgery. Obes. Surg..

[16] Edwards-Hampton S.A., Madan A., Wedin S., Borckardt J.J., Crowley N., Byrne K.T. (2014). A Closer Look at the Nature of Anxiety in Weight Loss Surgery Candidates. Int. J. Psychiatry Med..

[17] de Zwaan M., Enderle J., Wagner S., Mühlhans B., Ditzen B., Gefeller O., Mitchell J.E., Müller A. (2011). Anxiety and Depression in Bariatric Surgery Patients: A Prospective, Follow-Up Study Using Structured Clinical Interviews. J. Affect. Disorders.

[18] Kalarchian M.A., Marcus M.D., Levine M.D., Courcoulas A.P., Pilkonis P.A., Ringham R.M., Soulakova J.N., Weissfeld L.A., Rofey D.L. (2007). Psychiatric Disorders Among Bariatric Surgery Candidates: Relationship to Obesity and Functional Health Status. Am. J. Psychiatry.

[19] Mühlhans B., Horbach T., de Zwaan M. (2009). Psychiatric Disorders in Bariatric Surgery Candidates: A Review of the Literature and Results of a German Prebariatric Surgery Sample. Gen. Hosp. Psychiatry.

[20] Sarwer D.B., Cohn N.I., Gibbons L.M., Magee L., Crerand C.E., Raper S.E., Rosato E.F., Williams N.N., Wadden T.A. (2004). Psychiatric Diagnoses and Psychiatric Treatment Among Bariatric Surgery Candidates. Obes. Surg..

[21] Amiri S., Behnezhad S. (2019). Obesity and Anxiety Symptoms: A Systematic Review and Meta-Analysis. Neuropsychiatrie.

[22] Fulton S., Décarie-Spain L., Fioramonti X., Guiard B., Nakajima S. (2022). The Menace of Obesity to Depression and Anxiety Prevalence. Trends Endocrinol. Metab..

[23] Moroshko I., Brennan L., O’Brien P. (2011). Predictors of Dropout in Weight Loss Interventions: A Systematic Review of the Literature. Obes. Rev..

[24] Ponzo V., Scumaci E., Goitre I., Beccuti G., Benso A., Belcastro S., Crespi C., De Michieli F., Pellegrini M., Scuntero P. (2021). Predictors of Attrition from a Weight Loss Program: A Study of Adult Patients with Obesity in a Community Setting. Eat Weight Disord..

[25] Neri L.D.C.L., Mariotti F., Guglielmetti M., Fiorini S., Tagliabue A., Ferraris C. (2024). Dropout in Cognitive Behavioral Treatment in Adults Living with Overweight and Obesity: A Systematic Review. Front. Nutr..

[26] Perna S., Salman M., Gasparri C., Cavioni A., Faliva M.A., Mansueto F., Naso M., Patelli Z., Peroni G., Tartara A. (2022). Two, Six, and Twelve-Month Dropout Rate and Predictor Factors after a Multidisciplinary Residential Program for Obesity Treatment: A Prospective Cohort Study. Front. Nutr..

[27] Verhaak A.M., Ferrand J., Puhl R.M., Tishler D.S., Papasavas P.K., Umashanker D. (2022). Experienced Weight Stigma, Internalized Weight Bias, and Clinical Attrition in a Medical Weight Loss Patient Sample. Int. J. Obes..

[28] Miller B.M., Brennan L. (2015). Measuring and Reporting Attrition from Obesity Treatment Programs: A Call to Action!. Obes. Res. Clin. Pract..

[29] Paolino L., Le Fouler A., Epaud S., Bathaei S., Mokhtari N., Lazzati A. (2023). Preoperative Follow-up in Bariatric Surgery: Why They Give Up? Rate, Causes, and Economic Impact of Dropout. Obes. Surg..

[30] Gillieron E. (1989). Short Psychotherapeutic Interventions (Four Sessions). Psychother. Psychosom..

[31] Gilliéron E. (1994). Le Premier Entretien en Psychothérapie.

[32] Gilliéron E. (1997). Manuel de Psychothérapies Brèves.

[33] Derogatis L.R., Unger R. (2010). Symptom Checklist-90-Revised. The Corsini Encyclopedia of Psychology.

[34] Sarno I., Preti E., Prunas A., Madeddu F. (2011). SCL-90-R, Adattamento Italiano.

[35] Patton J.H., Stanford M.S., Barratt E.S. (1995). Factor Structure of the Barratt Impulsiveness Scale. J. Clin. Psychol..

[36] Reise S.P., Moore T.M., Sabb F.W., Brown A.K., London E.D. (2013). The Barratt Impulsiveness Scale–11: Reassessment of Its Structure in a Community Sample. Psychol. Assess..

[37] Fossati A., Di Ceglie A., Acquarini E., Barratt E.S. (2001). Psychometric Properties of an Italian Version of the Barratt Impulsiveness Scale-11 (BIS-11) in Nonclinical Subjects. J. Clin. Psychol..

[38] Gormally J.I.M., Black S., Daston S., Rardin D. (1982). The Assessment of Binge Eating Severity Among Obese Persons. Addict. Behav..

[39] Ricca V., Mannucci E., Moretti S., Di Bernardo M., Zucchi T., Cabras P.L., Rotella C.M. (2000). Screening for Binge Eating Disorder in Obese Outpatients. Compr. Psychiatry.

[40] Mannucci E., Ricca V., Barciulli E., Di Bernardo M., Travaglini R., Cabras P.L., Rotella C.M. (1999). Quality of Life and Overweight: The Obesity Related Well-being (Orwell 97) Questionnaire. Addict. Behav..

[41] Mannucci E., Ricca V., Barciulli E., Di Bernardo M., Travaglini R., Cabras P.L., Rotella C.M., Molinari E., Castelnuovo G. (2012). Obesity-Related WELL-being Questionnaire (ORWELL 97). Clinica Psicologica dell’Obesità.

[42] Butcher J.N. (2001). Minnesota Multiphasic Personality Inventory-2: Manual for Administration, Scoring, and Interpretation.

[43] Pancheri P., Sirigatti S. (1995). Minnesota Multiphasic Personality Inventory-2.

[44] Sirigatti S., Stefanile C. (2011). MMPI-2: Aggiornamento All’adattamento Italiano.

[45] Tellegen A., Ben-Porath Y.S. (1992). The New Uniform T-Scores for the MMPI-2: Rationale, Derivation, and Appraisal. Psychol. Assess..

[46] Cohen J. (1988). Statistical Power Analysis for the Behavioral Sciences.

[47] IBM Corp (2021). IBM SPSS Statistics for Windows.

[48] Hastie T., Tibshirani R., Friedman J.H. (2009). The Elements of Statistical Learning: Data Mining, Inference, and Prediction.

[49] Dwork C., Feldman V., Hardt M., Pitassi T., Reingold O., Roth A. (2015). The Reusable Holdout: Preserving Validity in Adaptive Data Analysis. Science.

[50] Dwyer D.B., Efallkai P., Koutsouleris N. (2018). Machine Learning Approaches for Clinical Psychology and Psychiatry. Annu. Rev. Clin. Psychol..

[51] Orrù G., Monaro M., Conversano C., Gemignani A., Sartori G. (2020). Machine Learning in Psychometrics and Psychological Research. Front. Psychol..

[52] Bermingham M.L., Pong-Wong R., Spiliopoulou A., Hayward C. (2015). Application of High-Dimensional Feature Selection: Evaluation for Genomic Prediction in Man. Sci. Rep..

[53] Kursa M.B., Rudnicki W.R. (2010). Feature Selection with the Boruta Package. J. Stat. Softw..

[54] Kohavi R. A Study of Cross-validation and Bootstrap for Accuracy Estimation and Model Selection. Proceedings of the 14th International Joint Conference on Artificial Intelligence.

[55] Kuhn M. (2008). Building Predictive Models in R Using the Caret Package. J. Stat. Softw..

[56] Chambers J.M., Hastie T.J. (1992). Statistical Models in S..

[57] le Cessie S., van Houwelingen J.C. (1992). Ridge Estimators in Logistic Regression. Appl. Stat..

[58] Breiman L. (2001). Random Forest. Mach. Learn..

[59] John G.H., Langley P. Estimating Continuous Distributions in Bayesian Classifiers. Proceedings of the 11th Conference on Uncertainty in Artificial Intelligence.

[60] Mitchell T., Mitchell T. (1997). Decision Tree Learning. Machine Learning.

[61] R Core Team (2021). R: A Language and Environment for Statistical Computing.

[62] Abbate L., Roma P. (2014). MMPI-2: Manuale per l’Interpretazione e Nuove Prospettive di Utilizzo.

[63] Zoccali R., Bruno A., Muscatello M.R., Micò U., Corica F., Meduri M. (2008). Defense Mechanisms in a Sample of Non-psychiatric Obese Subjects. Eat. Behav..

[64] American Psychiatric Association (2021). Diagnostic and Statistical Manual of Mental Disorders, Fifth Edition, Text Revision (DSM-5-TR™).

[65] Jensen H.H., Mortensen E.L., Lotz M. (2013). Scl-90-R Symptom Profiles and Outcome of Short-term Psychodynamic Group Therapy. Int. Sch. Res. Not..

[66] Van Hout G.C., Verschure S.K., Van Heck G.L. (2005). Psychosocial Predictors of Success Following Bariatric Surgery. Obes. Surg..

[67] Fidelix Y.L., Farias J.C.D., Lofrano-Prado M.C., Guerra R.L.F., Cardel M., Prado W.L.D. (2015). Multidisciplinary Intervention in Obese Adolescents: Predictors of Dropout. Einstein.

[68] Wu Y.K., Berry D.C. (2018). Impact of Weight Stigma on Physiological and Psychological Health Outcomes for Overweight and Obese Adults: A Systematic Review. J. Adv. Nurs..

